# Lower Dietary Inflammatory Index Scores Are Associated with Lower Glycemic Index Scores among College Students

**DOI:** 10.3390/nu10020182

**Published:** 2018-02-07

**Authors:** Yeonsoo Kim, Jie Chen, Michael D. Wirth, Nitin Shivappa, James R. Hebert

**Affiliations:** 1 Department of Human Environmental Studies, Central Michigan University, Mount Pleasant, MI 48858, USA; 2 School of Human Ecology, Louisiana Tech University, Ruston, LA 71270, USA; chenjiexychu@hotmail.com; 3 South Carolina Statewide Cancer Prevention and Control Program, University of South Carolina, Columbia, SC 29208, USA; wirthm@mailbox.sc.edu (M.D.W.); shivappa@mailbox.sc.edu (N.S.); jhebert@sc.edu (J.R.H.); 4 Department of Epidemiology and Biostatistics, University of South Carolina, Columbia, SC 29208, USA; 5 Connecting Health Innovations LLC, Columbia, SC 29201, USA; 6 College of Nursing, University of South Carolina, Columbia, SC 29208, USA

**Keywords:** dietary inflammatory index, glycemic index, glycemic load, healthy eating index-2010, college students

## Abstract

The association between the Dietary Inflammatory Index (DII^®^), the glycemic index (GI), and the glycemic load (GL) is not known, although it is known that carbohydrates are pro-inflammatory. We aimed to measure the association between the DII and both GI and GL among college students. In this cross-sectional study, 110 college students completed a 3-day food diary, which was used to calculate the DII, the GI, the GL, and the healthy eating index (HEI)-2010. Least square means and 95% confidence intervals of the GI, the GL, and the HEI-2010 were presented per DII tertile using generalized linear mixed models. Participants in tertile 1 of DII scores had lower GI and GL scores, but higher HEI-2010 scores than those in tertile 3. Pearson correlations showed that DII score was positively correlated with the GI score (*r* = 0.30, *p* < 0.01), but negatively correlated with the HEI-2010 (*r* = −0.56, *p* < 0.001). DII score was not correlated with GL score. Results from this study suggest that increased inflammatory potential of diet, as represented by higher DII scores, was associated with increased GI scores and lower quality of diet on the HEI-2010. Use of the DII suggests new directions for dietary approaches for preventing chronic diseases that moves beyond convention by decreasing systemic inflammation.

## 1. Introduction

Chronic low-grade inflammation is a major contributor to chronic diseases, including diseases such as diabetes and cardiovascular disease [1]. Although inflammation is a normal biological process needed for competent immune, vascular, and endothelial response, chronic inflammation can be caused by chronic infections that do not resolve and are associated with unhealthy lifestyle patterns, such as an unhealthy diet and tobacco use [2]. Given the significant effect of diet on developing inflammation and dietary modulation of the inflammatory cascade [3], diet has received special attention with respect to inflammation. The Dietary Inflammatory Index (DII^®^) was developed by researchers at the University of South Carolina specifically to measure the inflammatory potential of diet based on the overall inflammatory properties of dietary components, such as macronutrients, vitamins and minerals, flavonoids, and other bioactive compounds. The index classifies an individual’s diet on a continuum from maximally anti-inflammatory to maximally pro-inflammatory. The current DII is based on inflammation-focused, peer-reviewed literature and is standardized to the distribution of dietary intake from representative populations around the world [4].

In previous cross-sectional studies conducted in middle-aged adults and the elderly, the DII was validated against inflammatory biomarkers, such as C-reactive protein (CRP), interleukin-6 (IL-6), and tumor necrosis factor (TNF)-α [5,6,7,8,9,10,11]. Furthermore, the DII has been shown to be positively related to lower cognitive functioning [12] and higher risk of insulin resistance [13], cardiovascular disease (CVD) [11,14,15,16,17,18], and metabolic syndrome [15,19].

The glycemic index (GI) is a physiological carbohydrate-classification system based on glycemic response after consuming a carbohydrate-containing food compared to the response to a reference food, such as glucose solution or white bread containing the same amount of available carbohydrate [20]. The glycemic load (GL) is determined by multiplying a food’s GI by the amount of available carbohydrates in each serving size and dividing that number by 100 [21]. High GI foods, which are characteristically highly refined carbohydrates and/or carbohydrates with little fiber, are one of the major dietary factors affecting inflammation [22]. For example, cross-sectional studies reported a strong positive relationship between dietary GI [23] and GL [24] and plasma CRP in healthy, middle-aged women. Moreover, intervention studies showed that a low GI diet [25,26] and a low GL diet [27] lowered plasma CRP in short-term and long-term studies in overweight and obese adults.

Most DII studies have focused on middle-aged and elderly populations. To date, no DII study has compared DII scores with GI, GL and healthy eating index (HEI) scores among college students. A large body of research reports that most college students develop unhealthy eating habits, including excess consumption of calories, fat, refined grains, and processed foods [28,29,30,31,32,33]. In light of probable increased chronic disease risk resulting from college students’ unhealthy behaviors [34] and the crucial role of inflammation in developing chronic disease [16], it would be important to measure their DII scores and compare to GI, GL and HEI scores. It was hypothesized that college students will have pro-inflammatory diets (indicated by higher DII scores), and that higher DII scores will be associated with higher GI and GL scores, but lower HEI scores.

## 2. Materials and Methods

### 2.1. Subjects

Undergraduate students attending Louisiana Tech University (Ruston, LA, USA), which is located in the rural Southern USA, participated in this cross-sectional study from January 2013 to May 2013. Participants were recruited through advertising on campus. Potential participants were excluded if they were >29 years old, were on a diet, were pregnant, or had any chronic disease, such as diabetes mellitus, heart disease, or hypertension. This study was conducted according to the guidelines laid down in the Declaration of Helsinki. The study was approved by the Louisiana Tech University Human Subjects Review Committee. A signed consent form was obtained from each participant prior to the start of the study.

### 2.2. Data Collection

Dietary information was collected through a 3-day food diary. Participants were instructed to record their food intake for three days, including two non-consecutive weekdays and one weekend day. Food Processor Nutrition Analysis Software (ESHA Research, Salem, OR, USA) was used to analyze total calorie and nutrient intakes from the 3-day food diaries. Dietary data obtained after analyzing a 3-day food diary were used to calculate DII, GI, GL, and HEI-2010 scores.

To calculate DII scores, the method previously reported by Shivappa et al. [4] was used. In short, a literature review focusing on the association between food parameters and inflammatory markers was conducted to derive inflammatory effect scores for the food parameters. These scores were standardized to a world database, which includes a mean and standard deviation for each food parameter from 11 countries around the world. The mean value for each food parameter in the world database was subtracted from the actual reported intake of the food parameter, and then divided by the standard deviation to calculate a *Z*-score, which then was converted to a proportion (i.e., with values from 0 to 1) to minimize the effect of outliers. The proportion was then centered by doubling the proportion and subtracting 1. This value was multiplied by the inflammatory effect score for each food parameter. These were then summed across all food parameters to calculate the overall DII score. A higher DII score means greater pro-inflammatory potential of the diet. In this study the DII scores were calculated per 1000 calories consumed to account for inter-individual differences in energy intake [4]. The following 30 parameters were available to calculate the DII in the present study: carbohydrates, protein, total fat, alcohol, fiber, cholesterol, saturated fatty acids, trans fatty acids, monounsaturated fatty acids, polyunsaturated fatty acids, omega-3 fatty acids, omega-6 fatty acids, niacin, thiamine, riboflavin, vitamin B6, vitamin B12, vitamin A, vitamin C, vitamin D, vitamin E, folic acid, beta carotene, iron, magnesium, selenium, calcium, zinc, and caffeine. Energy (total caloric intake) was used as the divisor of the DII score to derive the energy-adjusted-DII (E-DII) score.

The GI values for carbohydrate-containing foods were extracted from the International Tables of GI and GL values for 2002 and 2008. The GI values in which glucose was employed as the reference food were used [35,36]. Each food had a number of GI values, and the selection of GI was made hierarchically in this order: (1) the same brand of food and method of preference; (2) a food produced in the USA; (3) average GI value of the same type of foods; or (4) otherwise closest match. The formula in which a meal GI was calculated from the individual food GI is as follows: Meal GI = {(GI_foodA_ × g available carbohydrate_foodA_) + (GI_foodB_ × g available carbohydrate_foodB_) + ∙∙∙}/total g available carbohydrate [37]. The GL values were calculated by multiplying the available carbohydrate amount in a serving size by its GI value, divided by 100 [37].

The HEI-2010, developed by the United States Department of Agriculture, was used to assess diet quality among the U.S. population. The HEI-2010 consists of 12 components, including nine adequacy components and three moderation components. The nine adequacy components, for which increased intake is recommended, are total fruit, whole fruit, total vegetables, greens and beans, whole grains, dairy, total protein foods, seafood and plant proteins, and fatty acids. In contrast, the three moderation components, for which decreased intake is recommended, include refined grains, sodium, and empty calories. Each component is proportionally scored from 0 to 10 based on minimum and maximum standards. The total score of the HEI-2010 ranges from 0 to 100 [38].

Weight and height of each participant were measured using a portable mechanical scale and stadiometer (SECA^®^, Road Rod 214, Hamburg, Germany), respectively. Weight was measured in light clothing and without shoes. Height was measured without shoes. Body mass index (BMI) was calculated by weight in kilograms divided by height in meters square (CDC). Waist circumference was measured at the midpoint between the lower ribs and the iliac crest using a non-stretchable measuring tape. Percent body fat was measured using the Tanita^®^ digital scale (TBF-300A, Tanita, Arlington Heights, IL, USA).

### 2.3. Statistical Analysis

Statistical Package for the Social Sciences (SPSS)^®^ software (Version 23.0, SPSS, Inc., Chicago, IL, USA) was used to perform the data analysis. Descriptive statistics included sex, race, smoking and drinking status, BMI classification, exercise status, age, BMI, percent body fat, and waist circumference. Descriptive statistics were conducted with chi-square tests for categorical variables and analysis of variance (ANOVA) tests for continuous variables. DII scores were converted into tertiles, and analyses were conducted using generalized linear mixed models, adjusting for age, sex, BMI, smoking status, drinking status, and physical activity level. The least squares means and 95% confidence intervals of the GI, the GL, and the HEI-2010 scores were presented per DII tertile using generalized linear mixed models. Pearson correlation coefficients were calculated to evaluate the association between the DII, the GI, the GL, and the HEI-2010 using the scores as continuous variables. Results were considered significant if the *p*-value of the analysis was <0.05.

## 3. Results

Participants’ demographic information is shown in Table 1. Fifty males (45%) and sixty females (55%) completed the study. The majority of the participants were European American (61.8%), non-smokers (88.2%), and non-drinkers (62.7%). Slightly more than half of the participants (52.7%) were normal weight, with an average BMI of 25.4 kg/m^2^. There were no significant differences on sex; race; smoking, drinking, and physical activity status; BMI; body-fat percentage; and waist circumference based on DII tertiles.

Participants’ intakes of nutrients and dietary components based on DII tertiles are provided in Table 2. Participants in the lowest DII tertile, indicating more anti-inflammatory diets, had higher consumption of total calories, monounsaturated fats, and all vitamins and minerals than individuals in the highest DII tertile, whose diets were more pro-inflammatory. In contrast, college students in the lowest DII tertile consumed lower amounts of total fat and saturated fat than participants in the highest DII tertile (*p* = 0.05 and <0.001, respectively).

College students in the lowest DII tertile had higher scores for the HEI-2010 than those in the highest DII tertile (50.7 vs. 35.4, *p* < 0.001). However, participants in the DII tertile 1 had lower scores for the GI than those in DII tertile 3 (50.1 vs. 54.8, *p* = 0.02). Only females in the lowest DII tertile had lower scores for the GI than those in the DII tertile 3 (47.5 vs. 53.8, *p* = 0.02) (Table 3). DII score was positively correlated with GI score (*r* = 0.30, *p* < 0.01), but negatively correlated with the HEI-2010 score (*r* = −0.56, *p* < 0.001). DII score was not related to the GL (*r* = −0.03, *p* = 0.76) (Table 4).

In this present study, among college students residing in the rural Southern US, the ten most popular food items, based on frequency of consumption, included French fries, wheat bread, lemonade, pepperoni pizza, chicken sandwich, sweet tea, chicken nugget, apple, banana, and Cajun style rice, in descending order (Table 5).

## 4. Discussion

The present study found that DII score was positively associated with GI score, but negatively associated with the HEI-2010 among rural, Southern US college students. Previous studies that measured the association between the DII and the HEI-2010 among participants 21 to 35 years of age, also reported an inverse relationship between the DII and the HEI-2010 [39,40,41].

Studies have reported the mechanism by which high GI diets induce inflammation. High GI diets induce hyperglycemia, which in turn induces oxidative stress [42] and increases pro-inflammatory cytokines, including IL-6 and TNF-α, among both healthy subjects and those with impaired glucose tolerance [43]. The increased level of pro-inflammatory cytokines, in turn, causes disruptions in insulin signaling, subsequently leading to insulin resistance [44]. Furthermore, insulin-resistance-related hyperglycemia causes accumulation of advanced glycation end products, which increase production of pro-inflammatory cytokines in vascular endothelial cells [45]. Altered endothelial permeability and reduced blood flow may augment insulin resistance, resulting in exacerbating hyperglycemia, leading to a vicious cycle of reduced blood flow and insulin resistance [46].

Given the impact of hyperglycemia on inflammatory pathways, achieving normoglycemia has been recognized as key to improving outcomes and minimizing pro-inflammatory mechanisms. Studies have shown a positive association between dietary GI and GL and inflammation biomarkers [22,23] because a low GI diet reduces the rate of glucose absorption by the body, subsequently diminishing hyperglycemia and hyperinsulinemia, which then induce a reduction in systemic inflammation. For example, a low GI [47,48] or low GL diet [27,49,50] resulted in weight loss in overweight and obese subjects, along with improvements in insulin sensitivity. The weight loss also caused reduction in pro-inflammatory cytokines. Considering the recognized benefits of a low-GI diet, it has been recommended by various health organizations to prevent or manage diabetes and coronary heart disease, and a likely weapon against obesity [51,52].

GI scores have been reported to be associated inversely with HEI-2010 scores [53,54]. The HEI-2010 is a measure of diet quality to assess adherence to the 2010 Dietary Guidelines for Americans and to describe Americans’ [38] eating patterns. Moreover, the HEI-2010 assesses the healthfulness of the overall diet, as the index captures all dietary components, rather than only selected nutrients or food groups [38]. According to the results of the National Health and Nutrition Examination Survey (NHANES) 2007–2010, the average HEI-2010 score among U.S. adults ages 20 to 29 was 48.8 (95% confidence interval (CI) 47.2, 50.5), while the average score among all U.S. adults was 55.9 (95% CI 54.5, 57.3) [55]. In the present study, the average HEI-2010 score was 43.6, which is slightly lower than the national average of young adults in their 20s. Given that a diet with a score of less than 51 is considered poor [38], the overall diet quality of the college students participating in the present study seems poor. It is well known that college students’ eating habits fall far short of general dietary recommendations. For example, they tend to skip meals frequently, eat few servings of fruits and vegetables, and consume too many calories and too much total fat [33]. Furthermore, the ten most popular foods among college students fit typical Western dietary patterns, with sweetened beverages, candies, instant noodles, refined grains, and pizza among the top ten [33,45]. The Western pattern had a positive association with CRP after adjustment for all confounding factors, including BMI [45].

Although values for U.S. college students could not be separated from those of others, the mean dietary GI and GL of adults in the NHANES 2003–2006 were 56.2 and 138.1, respectively [56]. In the current study, the average dietary GI and GL were 52.3 and 158.0, respectively. Based on the definition of low-GI foods (GI value 55 or less) [57], 5 out of top ten popular food items were classified as low GI foods. Wheat bread and pepperoni pizza were the 2nd and the 4th most popular-foods, respectively, in the present study and their GI values were a little higher than 55, which is the cut-off point for low GI foods. Including fruits and wheat bread in the list of their popular foods consumed by the participants would explain the relatively low GI value in this group. Due to the Southern culture, sweet tea and Cajun-style rice were ranked as 6th and 10th, respectively, in this population. Although some of the popular items listed are high-fat foods, such as French fries, their GI scores are not high. The GI scores are affected by the amount of fat and protein [58]. A study showed that consuming a low GI diet for 30 days reduced body fat and TNF-α in patients with type 2 diabetes when both high GI and low GI diets had similar energy density, dietary fiber, and macronutrient contents [59].

In middle-aged American women, cooked potato, cold breakfast cereal, and white bread were, the major contributors to dietary GL [58]. In older Australian women, white bread, potatoes, breakfast cereals, and fruit, specifically banana, contributed to dietary carbohydrate and GL significantly [24]. In the present study conducted in Southern US college students, French fries and wheat bread were major contributor to GL. The GL can be reduced by consumption of a diet high in low-GI carbohydrates or a diet low in carbohydrates and high in protein and fat. In trying to achieve acceptable macronutrient distribution range and a high-quality diet, it would be reasonable to recommend smaller portions of less healthy high-GI foods and larger portions of low-GI foods [60].

In this study the correlation coefficient of 0.30 between DII score and GI score is much lower than the absolute values of correlation coefficients that we have observed between the DII score and other indices in other studies [39,40,41]. This is revealing in that less than 10% of the variability in the DII score is explained by the GI score. This is reasonable on its face because simple carbohydrates are just one of the contributors to the DII score. The implication that factors that are associated with high glycemic load account for only a small fraction of the total inflammatory capacity of diet, is important.

Because there has been increased research interest in inflammation’s effect on health, a diet plan emphasizing a selection of foods that reduce inflammatory pathways while discouraging consumption of foods that promote inflammation is ideal [26]. Specifically, an anti-inflammatory diet contains fruits and vegetables with high concentrations of fiber, vitamins, minerals, and polyphenols, as well as herbs and spices. Moreover, most fruits and vegetables have low glycemic indexes because of high amounts of fiber [27]. Considering the characteristics of an anti-inflammatory diet plan, the DII was developed specifically to measure the inflammatory potential of any diet [4]. Numerous studies showed that the DII predicts concentrations of inflammatory biomarkers, including CRP, IL-6, TNF-α, etc., in cross-sectional and longitudinal studies [7,8,10,13,61].

Considering the positive association between the DII and inflammation biomarkers, the DII appears to be an excellent tool to determine dietary inflammatory potential [8]. The DII also suggests new directions for nutrition education and dietary approaches for preventing chronic diseases by decreasing systemic inflammation. Several factors affect the development of systemic inflammation, including age, genetic makeup, obesity, and diet. Among contributing factors, diet is critical because it can be changed [12].

One strength of the present study is that it is the first study in which DII, GI, and GL were measured among college students. In light of the importance of developing good eating habits in early adulthood, the present study’s results showed the current status of dietary inflammatory potential and the need for college students to improve their diets. Furthermore, a 3-day food diary was used to assess dietary intake to minimize day-to-day variations. Another strength is the relative sex balance in participants, at 45% male and 55% female, which enabled data analysis stratified by sex.

Despite the strengths of the study, several limitations must be considered. First, because of the current study design, the temporality criterion of the Criteria for Judging Causality is not met [62]. Hence, direct causal inference could not be made. Second, college students attending a rural, public, Southern U.S. University participated in the present study; so, caution is needed when extrapolating study results to other populations. Third, because participants recorded their 3-day food diary, there might have been reporting errors. However, to minimize any errors, participants were interviewed by the principal investigator upon completion of their food diaries to confirm their food consumption.

The DII was developed to assess dietary inflammatory potential. Of several studies that have examined these relationships, virtually all have observed a positive association between DII score and inflammation biomarkers (though, admittedly, there is variability across studies and across markers) [7,8,41,61,63,64,65]. Although the DII can be used successfully in nutritional studies, it might be challenge for laypersons to use to measure their dietary inflammatory potential on their own, without technical assistance (i.e., providing web or other technology-based tools for dietary assessment). Given that a low-GI diet reduces inflammatory responses and the present study reported an association between DII and GI scores, the GI may be used as very crude tool to provide a rough estimate of dietary inflammatory response.

## 5. Conclusions

In conclusion, the results of the present study suggest an inverse association between the DII and dietary GI among college students. The results of the present study also are in line with epidemiological findings on the association between dietary GI and HEI, a widely used dietary index. Given deepening understanding of the role of inflammation in a wide variety of health conditions [11,14,15,16,17,19,65], aging [63] and cognitive decline [12], the DII represents a potential tool for clinical or even individual use. However, this will require that individuals are provided with the necessary technical tools to compute scores from their dietary self-reports.

## Figures and Tables

**Table 1:** Participants’ characteristics by Dietary Inflammatory Index (DII) tertiles.

	Total (*n* = 110)	DII Tertile 1 <2.8 (*n* = 37)	DII Tertile 2 2.8–4.6 (*n* = 37)	DII Tertile 3 >4.6 (*n* = 36)	*p*-Value
Sex					0.25
Male	50 (45.5%)	20 (54.1%)	13 (35.1%)	17 (47.2%)	
Female	60 (54.5%)	17 (45.9%)	24 (64.9%)	19 (52.8%)	
Race					0.21
European American	68 (61.8%)	28 (75.7%)	22 (59.5%)	18 (50.0%)	
African American	36 (32.7%)	8 (21.6%)	12 (32.4%)	16 (44.4%)	
Asian	6 (5.5%)	1 (2.7%)	3 (8.1%)	2 (5.6%)	
Smoker					0.26
No	97 (88.2%)	34 (91.9%)	30 (81.1%)	33 (91.7%)	
Yes	13 (11.8%)	3 (8.1%)	7 (18.9%)	3 (8.3%)	
Drinker					0.81
No	69 (62.7%)	23 (62.2%)	22 (59.5%)	24 (66.7%)	
Yes	41 (37.3%)	14 (37.8%)	15 (40.5%)	12 (33.3%)	
BMI classification ^1^					0.38
Normal	58 (53.2%)	17 (47.2%)	21 (56.8%)	20 (55.6%)	
Overweight	32 (29.4%)	11 (30.6%)	13 (35.1%)	8 (22.2%)	
Obese	19 (17.4%)	8 (22.2%)	3 (8.1%)	8 (22.2%)	
Exercise					0.42
No	18 (16.4%)	4 (10.8%)	6 (16.2%)	8 (22.2%)	
Yes	92 (83.6%)	33 (89.2%)	31 (83.8%)	28 (77.8%)	
Age (years)	21.0 ± 2.5	21.3 ± 2.7	21.4 ± 3.0	20.0 ± 1.4	0.09
BMI (kg/m^2^)	25.4 ± 4.6	25.9 ± 4.7	24.8 ± 3.5	25.3 ± 5.6	0.61
Body fat (%)	21.3 ± 9.4	20.6 ± 10.0	22.7 ± 8.9	20.7 ± 9.4	0.57
Waist circumference (cm)	81.2 ± 11.8	82.5 ± 10.9	80.0 ± 9.0	81.0 ± 15.0	0.67

Values are expressed as mean ± standard deviation (SD). ^1^ *n* = 109. BMI: body mass index.

**Table 2:** Nutrient intake of participants by DII tertiles.

	DII Tertile 1 <2.8 (*n* = 37)	DII Tertile 2 2.8–4.6 (*n* = 37)	DII Tertile 3 >4.6 (*n* = 36)	*p*-Value
Calories (cal)	2674.2 ± 778.9	2227.1 ± 778.9	2499.3 ± 778.9	0.05
Carbohydrates (g)	300.3 ± 67.9	296.1 ± 68.2	292.3 ± 31.1	0.88
Protein (g)	105.4 ± 31.5	98.4 ± 31.7	87.4 ± 67.1	0.05
Total fat (g)	92.8 ± 21.0	97.8 ± 21.6	105.2 ± 21.3	0.05
Saturated fat (g)	27.2 ± 8.42	29.3 ± 8.4	35.7 ± 8.3	<0.001
Monounsaturated fatty acid (g)	17.8 ± 9.7	16.5 ± 9.7	12.0 ± 9.5	0.03
Polyunsaturated fatty acid (g)	9.2 ± 7.4	9.9 ± 7.4	6.2 ± 7.3	0.08
Cholesterol (mg)	362.7 ± 306.2	394.5 ± 307.5	408.1 ± 302.3	0.81
Trans fat (g)	1.5 ± 2.5	1.9 ± 2.5	2.8 ± 2.5	0.09
Vitamin A (RE)	664.9 ± 474.8	305.8 ± 476.9	189.5 ± 468.8	<0.001
Vitamin B1 (mg)	1.3 ± 0.5	0.9 ± 0.5	0.6 ± 0.5	<0.001
Vitamin B2 (mg)	2.2 ± 1.1	1.4 ± 1.1	0.8 ± 1.1	<0.001
Niacin (mg)	28.1 ± 9.8	21.2 ± 9.8	11.2 ± 9.7	<0.001
Vitamin B6 (mg)	1.9 ± 0.9	1.2 ± 0.9	0.7 ± 0.8	<0.001
Vitamin B12 (μg)	6.0 ± 3.3	4.0 ± 3.3	1.6 ± 3.3	<0.001
Vitamin C (mg)	101.8 ± 62.8	75.5 ± 63.1	45.7 ± 62.0	0.001
Vitamin D (IU)	3.8 ± 3.1	2.2 ± 3.1	1.3 ± 3.1	0.004
Vitamin E -α-tocopherol (mg)	6.6 ± 4.5	3.3 ± 4.5	1.4 ± 4.4	<0.001
Folate (μg)	337.1 ± 118.8	218.7 ± 119.4	123.1 ± 117.3	<0.001
Vitamin K (μg)	99.9 ± 91.7	20.3 ± 92.3	15.9 ± 91.9	<0.001
Calcium (mg)	1064.3 ± 381.9	738.8 ± 383.6	685.9 ± 377.1	<0.001
Iron (mg)	18.3 ± 6.9	17.1 ± 6.9	10.9 ± 6.8	<0.001
Magnesium (mg)	218.3 ± 64.3	120.1 ± 64.6	73.3 ± 63.5	<0.001
Phosphorus (mg)	963.8 ± 361.9	715.9 ± 363.5	500.5 ± 357.3	<0.001
Potassium (mg)	1942.4 ± 591.6	1346.1 ± 594.3	890.2 ± 584.2	<0.001
Selenium (μg)	77.2 ± 45.5	62.3 ± 45.6	25.7 ± 44.9	<0.001
Sodium (mg)	4100.3 ± 1154.5	4493.8 ± 1159.9	4746.9 ± 1140.0	<0.057
Zinc (mg)	8.0 ± 3.2	6.0 ± 3.2	3.4 ± 3.2	<0.001
Caffeine	66.7 ± 79.6	53.7 ± 79.9	45.0 ± 79.7	0.50

Values are expressed as mean ± standard deviation (SD). Values are adjusted for energy, age, and sex.

**Table 3:** Healthy eating index (HEI)-2010, Glycemic index, and Glycemic load by DII tertiles.

Dietary Indices	DII Tertile 1 <2.8 (*n* = 37)	DII Tertile 2 2.8–4.6 (*n* = 37)	DII Tertile 3 >4.6 (*n* = 36)	*p*-Value
Total				
Glycemic Index (GI)	50.1 (47.9–52.3)	51.9 (49.7–54.0)	54.8 (52.5–57.0)	0.02
Glycemic Load (GL)	170.7 (149.4–192.0)	145.8 (125.0–166.6)	157.6 (136.0–179.3)	0.27
Healthy Eating Index (HEI)-2010	50.7 (47.3–54.0)	44.6 (41.3–47.9)	35.4 (32.0–38.8)	<0.001
Male				
GI	52.5 (49.1–55.9)	53.9 (49.4–58.3)	56.7 (52.7–60.8)	0.30
GL	167.5 (135.8–199.2)	174.7 (135.2–214.2)	185.0 (148.8–221.2)	0.79
HEI-2010	48.1 (43.2–53.0)	39.9 (33.4–46.4)	29.5 (24.1–35.0)	<0.001
Female				
GI	47.5 (44.3–50.6)	50.0 (47.5–52.4)	53.8 (51.0–56.7)	0.02
GL	149.8 (130.3–169.2)	137.4 (121.9–152.9)	146.4 (128.0–164.8)	0.56
HEI-2010	55.6 (50.5–60.7)	46.8 (42.8–50.8)	39.1 (34.3–43.9)	<0.001

Values are expressed as means and 95% confidence intervals of the HEI-2010, GI, and GL. Values are adjusted for age, race, sex, BMI, percent body fat, smoker, drinker, and physical activity level.

**Table 4:** Correlation between DII and HEI, Glycemic index (GI), and Glycemic load (GL).

	Total		Males		Females	
	*r*	*p*-Value	*r*	*p*-Value	*r*	*p*-Value
DII-GI	0.30	<0.01	0.27	0.07	0.32	0.02
DII-GL	−0.03	0.76	−0.15	0.34	0.14	0.30
DII-HEI	−0.56	<0.001	−0.54	<0.001	−0.60	<0.001
HEI-GI	−0.47	<0.001	−0.49	0.01	−0.46	<0.001
HEI-GL	−0.43	<0.001	−0.46	0.01	−0.44	0.01

Values are adjusted for age, race, sex, BMI, percent body fat, smoker, drinker, and physical activity level.

**Table 5:** Ten most popular food items.

	Food Item	GI Value	Reference
1	French fries	64	#1658 from International Table of GI and GL values, 2008 [36]
2	Wheat bread	58	#96 from International Table of GI and GL values, 2002 [35]
3	Lemonade	54	#50 from International Table of GI and GL values, 2008 [36]
4	Pepperoni pizza	60	#488, cheese pizza from International Table of GI and GL values, 2002 [35]
5	Chicken sandwich	66	#1178, McChicken^TM^ burger from International Table of GI and GL values, 2008 [36]
6	Sweet tea	65	#589 from International Table of GI and GL values, 2002 [35]
7	Chicken nuggets	46	#482 from International Table of GI and GL values, 2002 [35]
8	Apple	40	#388, apple (USA) from International Table of GI and GL values, 2002 [35]
9	Banana	42	#397, Banana, slightly underripe, yellow with green sections (USA) from International Table of GI and GL values, 2002 [35]
10	Cajun-style rice	51	#281 from International Table of GI and GL values, 2002 [35]
